# A Short Sermon

**Published:** 1854-07

**Authors:** 


### ART. III.—*A Short Sermon.* By Clericus, Jr.

"And had suffered many things of many physicians, and had spent all that she had, and was nothing bettered, but rather grew worse."

My friends, this is a very impressive, and important text. It is full of meaning, point, and importance, particularly in so far as the medical profession are concerned.

I shall remark, 1st, This text proves the *antiquity* of the medical profession. It is from a very old, and, in the eyes of many, a very unfashionable book. Eighteen hundred years have rolled away since the important truths uttered in our text were made known to man. Such a lapse of time savors of antiquity; and a calling thus alluded to must be ancient, venerable, time-honored. Ancient things are invested with a sort of sacredness. Even an aged man commands respect. His bent form, his trembling limbs, his hoary locks, invest him with peculiar interest. So of the ancient profession of medicine. It smells of antiquity. You see antiquity written in the visage of many physicians of the present day. You see it in their dignified consequential bearing, in a certain air of loftiness, which is permeated with the essence of the past. Candor, however, compels me to add, that this dignified, ancient manner, smells of Old Fogyism—looks with contempt on modern innovations, and even goes so far as to prefer the death of a patient under the old regime, rather than his recovery under the new.

Secondly. My text proves that physicians were *numerous* in those days,—hence the phrase, "many physicians." It is devoutly to be hoped, that as a class, they were many degrees in advance of the profession of the present day. Even now they cover the whole land; they eat up every green thing; they prowl around our houses, enter our very bed-chambers, and, if our kneading troughs were of sufficient capacity, no doubt they would fill them also. What an army! clothed in a glorious panoply: to wit—knives, lancets, blood-suckers, blisters! What heroic doses of calomel, jalap, scammony and gamboge! What a nauseating smell of ether and assafœtida! What a capacious armamentarium of remedies from the heavens above, (electricity,) from the earth beneath, (instruments of steel and iron,) from even the waters under the earth. Truly, this is a goodly company and strong. Death surely ought to flee away from mere fright.

Thirdly. My text says, that these many physicians had a patient, and that the patient was very sick. This, my brethren, is by no means strange. It so happens, at the present day. Every tatterdemalion and fool who dubs himself M. D., has his patients, no matter how slim his abilities, how empty his brain. The world is full of fools, and you cannot blame them, surely, when they trust so insignificant a thing as life in the hands of men whose intellectual capacity and sense is on a par with their own. As I said before, this patient was very sick, and employed a vast number of physicians. They, doubtless, did their best. They ransacked the earth, and dealt out doses in quality, quantity, and power, such as "ancient physicians" only know how to give.

Fourthly. I remark that this patient *suffered,* not once or twice, but much. The text says that she "suffered many things," which means, I suppose, that she took many and large doses. Now, this short sentence, apparently so insignificant, knocks homœopathy, vulgarly speaking, into a cocked hat. It strips the unholy bantling of all the dignity which belongs to an old time-honored calling. Now, these physicians alluded to, could not have been the disciples of befooled, besotted, and besmoked Hahnemann. This woman, remember, *suffered.* She, doubtless, groaned in body and spirit. Her feeble stomach revolted at the enormous quantities of "unmitigated nastiness" which were forced down without stint. Now, under homœopathic treatment, it is perfectly preposterous to suppose that she endured anything. What amount of suffering is there in taking sugar comfits? How much would any one endure in swallowing attenuated doses, reduced in quantity and effect to a nonentity? I pause for an answer. Why, my brethren, it would have been a pleasure, rather than a suffering, to have taken the infinitesimals prescribed by those smooth, insinuating, long-eared gentry, with oil on their hair and on their tongues, so much in request by the fools of the present day. I wish to have you understand, that it is the regular profession, and the regular profession only, that are worthy of notice in those early days. Men then believed in something tangible, and if they did not get better, they must certainly get worse under medical treatment.

Fifthly. I remark, that "she had spent all that she had." The poor soul sought every available means of relief, and though her disease went on unchecked, she was effectually relieved of her cash. Yes, *all;* not a single stiver was left. Those vampires, not satisfied with drawing her blood, must also draw all of her property. She, it seems, did not complain of robbery and extortion. She rather expected it, and her expectation was fulfilled to the very letter. She was a sensible woman. She, doubtless, was well aware that the ancient and dignified profession was not insensible to the value of ready money, and she submitted with good grace. My brethren, these hard-hearted, callous, avaricious souls, exist even at the present day. Every pang is to them duly estimated worth so much in the market. Every groan has a money value. When the day of hope is past, and the grave opens wide —when sorrowing dependents and relatives gather around, *their* anxiety concerns principally the bill "for services rendered." I sincerely hope, my friends, that in modern times, there may be, even in the medical profession, men with hearts as large, free and sympathetic, as there are woes bitter and heart-rending in the depths of human suffering.

Sixthly, and lastly. I remark, "that she was nothing bettered, but rather grew worse." This is no strange result. Men, and women too, get worse every day under the regular treatment. Why, my friends, I had rather have a band of regular physicians, and ancient physicians too, for execution, than the best drilled brigade of soldiers that will be marshaled in the present Eastern war. They couldn't kill, if they should try their best, as surely and quickly as the faculty. Anybody can load and fire a gun, but they cannot do the work with the cool sang froid of an ancient physician. I could stand here all day and enumerate examples where ancient physicians have done terrible execution. Not a week ago, some of these ancient physicians killed a patient laboring under peritonitis, by dosing her with drastic cathartics. Now, my friends, I do not believe in this heroic dosing, unless you are certain of a cure. I know that when I give my peritonitic patients cathartics, they always die.

*Improvement.* We see from this subject, that these ancient physicians cut rather a sorry figure. From the history of the disease in the context, we cannot infer, with any degree of certainty, whether the complaint in question was curable under any circumstances, which is, indeed, a palliating thought. At any rate, however incurable the disease, there should have been, under judicious treatment, a temporary check.

We learn, also, that when "ancient" methods of treatment fail, we must rely upon an enlarged experience, a careful observation, a judicious prudence, and a sound philosophy, for some new method of cure. The experience of the past is a glorious legacy; but it does not equal in value the careful researches and developments of these latter days.

We also learn from this subject the necessity of humanity, kindness and benevolence in all our dealings with those under our care. We should not take advantage of their follies, their whims, or weaknesses, for our own profit. *Our* business is to do good, to tell the truth at all times, and when we find a case beyond human effort, to say so, and prepare the sufferer for the worst. We must eschew quackery, double dealing, and meanness, then we shall be a blessing, and we shall rise to a well merited position of dignity and usefulness. So mote it be.

T. H. S. M.

